# Neuroprotective potential of intranasally delivered L-myc immortalized human neural stem cells in female rats after a controlled cortical impact injury

**DOI:** 10.21203/rs.3.rs-3242570/v1

**Published:** 2023-09-05

**Authors:** Mari Amirbekyan, Jeffrey P. Cheng, Vikram Adhikarla, Eleni H. Moschonas, Corina O. Bondi, Russell C. Rockne, Anthony E. Kline, Margarita Gutova

**Affiliations:** Beckman Research Institute, City of Hope; University of Pittsburgh School of Medicine Children’s Hospital of Pittsburgh John G. Rangos Research Center – Room 6126; Beckman Research Institute, City of Hope; University of Pittsburgh School of Medicine Children’s Hospital of Pittsburgh John G. Rangos Research Center – Room 6126; University of Pittsburgh School of Medicine Children’s Hospital of Pittsburgh John G. Rangos Research Center – Room 6126; Beckman Research Institute, City of Hope; University of Pittsburgh School of Medicine Children’s Hospital of Pittsburgh John G. Rangos Research Center – Room 6126; Beckman Research Institute, City of Hope

## Abstract

Efficacious stem cell-based therapies for traumatic brain injury (TBI) depend on successful delivery, migration, and engraftment of stem cells to induce neuroprotection. L-myc expressing human neural stem cells (LMNSC008) demonstrate an inherent tropism to injury sites after intranasal (IN) administration. We hypothesize that IN delivered LMNSC008 cells migrate to primary and secondary injury sites and modulate biomarkers associated with neuroprotection and tissue regeneration. To test this, immunocompetent adult female rats received a controlled cortical impact injury (CCI) or sham surgery. LMNSC008 cells or a vehicle (VEH) were administered IN on postoperative days 7, 9, 11, 13, 15, and 17. The distribution and migration of eGFP-expressing LMNSC008 cells were quantified over 1 mm-thick optically cleared (CLARITY) coronal brain sections from TBI and SHAM controls. NSC migration was observed along white matter tracts projecting toward the hippocampus and regions of TBI. ELISA and Nanostring assays revealed a shift in tissue gene expression in LMNSC008 treated rats relative to controls. LMNSC008 treatment reduced expression of genes and pathways involved in inflammatory response, microglial function, and various cytokines and receptors. The data demonstrate a robust proof-of-concept for LMNSC008 therapy for TBI and provides a strong rationale for IN delivery for translation in TBI patients.

## Introduction

Traumatic brain injuries (TBIs) impact upwards of 10 million individuals worldwide each year[1], with approximately 2.8 million incidents reported yearly in the United States[2]. Depending on injury severity, the degree of tissue loss and damage to neuronal networks can be significant and result in transient to persistent cognitive and motor complications[3–5]. Empirical research investigating the pathology of TBI has yielded important insights regarding potential targets to manipulate for subsequent recovery from TBI. Unfortunately, despite the enthusiasm to develop therapeutics for TBI, of which pharmacotherapies make up the majority, none have consistently resulted in patient improvement[6].

The lack of FDA approved pharmacological interventions for TBI serves as the impetus for investigating its potentially beneficial approaches[7]. Neural stem cells (NSCs) are multipotent cells that can proliferate *in vitro* and differentiate *in vitro* and *in vivo* into multiple neuronal lineages[8–10]. We have developed and characterized allogeneic human NSCs genetically modified to express human L-myc gene (LMNSC008)[11]. These LMNSC008 cells can differentiate into neurons and glial cells *in vitro and in vivo*[11, 12]. NSCs have been shown to contribute to recovery from TBI via cell replacement and neuroprotective effects[9, 13–20]. However, much is still to be learned from this approach. Understanding the trajectory of NSCs in the TBI brain will enhance our understanding of how specialized stem cell treatments function in this context to improve recovery[20–25].

Hence, the overall goals of this study are to first delineate the migration patterns of intranasally (IN) delivered LMNSC008 cells and second to investigate how the cells change the milieu at TBI sites. The hypothesis is that LMNSC008 cells will migrate toward injury sites, where they will accumulate and contribute to recovery from TBI by reducing immune and inflammatory cytokine responses and improving angiogenesis.

## Materials and Methods

### Animals

All animal studies were performed according to ARRIVE (Animal Research: Reporting In Vivo Experiments) guidelines published in 2010 (https://arriveguidelines.org). Adult female Sprague-Dawley rats (n = 24) (Envigo RMS, Inc., Indianapolis, IN) were pair-housed in ventilated polycarbonate rat cages and maintained in a temperature (21 ± 1°C) and light (0700 h to 1900 h) controlled vivarium with *ad libitum* food and water. The rats were allowed to acclimate to the facility for one week before undergoing experimental procedures. After the acclimatization period, the rats were randomly assigned to the following groups: CCI + LMNSC008 (n = 8), CCI + VEH (n = 8). Control groups include SHAM + LMNSC008 (n = 4), and SHAM + VEH (n = 4) to assess experimental differences as compared with shame surgery groups +/− LMNSC008 cells [“LMNSC008” is shortened to “NSC” throughout this paper]. All experimental manipulations were conducted during the lights-on phase. All procedures were conducted in accordance with the *Guide for the Care and Use of Laboratory Animals* and were reviewed and approved by the University of Pittsburgh Institutional Animal Care and Use Committee (IACUC). Mindful effort was made to minimize animal pain, suffering, or discomfort, and to limit the number of rats used.

### Surgery

Anesthesia was induced with 4% isoflurane in 2:1 N2O:O2, which was followed by endotracheal intubation, placement on a stereotaxic frame, and mechanical ventilation. Surgical anesthesia was maintained with 2% isoflurane and the same volume of carrier gases. Core temperature was monitored and maintained at 37 ± 0.5°C with a rectal thermistor and heating pad, respectively. Utilizing aseptic procedures, a controlled cortical impact (CCI) injury was produced as previously described.[26, 27] Specifically, a midline scalp incision was made, the skin and fascia were opened, and a craniectomy was made in the right hemisphere with a high-speed dental drill. The bone flap was removed, and the craniectomy was enlarged further to fit the impact tip (6 mm, flat), which was lowered through the craniectomy until it touched the dura mater and then advanced farther to produce a brain injury of moderate severity (2.8 mm tissue deformation at 4 m/s). Sham rats were not subjected to the impact but did receive all other surgical manipulations to control for the potential effects of the anesthesia and craniectomy. After the injury procedures, the anesthesia was discontinued, the incision was promptly sutured closed, and the rats were extubated and assessed for acute neurological outcomes.

### Cyclosporin A, NSCs, and VEH administration

To enhance the engraftment of LMNSC008 cells, an immunosuppressive regimen of cyclosporin A (CsA; 10 mg/kg; s.c.) was provided to *all rats* beginning two days before the first IN administration of NSCs or VEH and continuing once per day until the brains were harvested.

Human LMNSC008 cells from our Master Cell Bank were genetically modified using lentivirus to express green fluorescent protein (eGFP) and firefly luciferase (FFluc) genes were prepared for IN administration according to established SOPs[12, 20, 28, 29]. Briefly, LMNSC008 cells were thawed, washed, and re-suspended in PBS (1×106 cells in 24 µL). Rats received IN injections (4 µL per drop and 3 drops per nare at 2 min intervals) for a total of 24 µL of LMNSC008 cells or the same volume of vehicle (VEH), which consisted of 2% HSA in PBS 20 min after disruption of the nasal epithelium with hyaluronidase. IN dosing occurred on days 7,9,11,13,15, and 17 after CCI or SHAM injury.

### Euthanasia and brain harvesting

39 days after CCI or sham injury, the rats were deeply anesthetized with Fatal-Plus^®^ (0.3 mL, i.p., Henry Schein Animal Health; Warrendale PA) and perfused transcardially with 300 mL of ice cold 0.1 M PBS and 600 mL of 4% paraformaldehyde (PFA). Brains were harvested and stored in PBS at 4°C until ready for the next experimental phase.

### Brain clearing

Harvested brains were cleared using a modified CLARITY-PACT passive tissue clearing protocol[30]. Briefly, the brains were trimmed with a razor to remove the olfactory bulbs and cerebellum, and then 8–10 coronal sections (1 mm thick) were cut with a vibratome and labeled (S1-S7) with S1 and S7 being most anterior and posterior to the CCI site, respectively. Slices were stored at 4°C overnight in PBS until processed for clearing and staining as described below. PFA-fixed brain sections were transferred to individual 50 mL conical tubes and infused with ice-cold A4P0 hydrogel solution (40% wt/vol acrylamide, 10X PBS, 0.25% wt/vol thermo initiator, dH20) for 24 h at 4°C. Unused A4P0 was stored at −20°C, and aliquots were thawed overnight at 4°C as needed (always protected from light). Each conical tube was topped with a rubber stopper, and the next day oxygen was depleted from the tubes using a vacuum line and venting needle inserted through the rubber stopper for 10 min. The conical tubes were then placed in a 37°C water bath for 2 h or until the hydrogel solution had polymerized into a gel or thick liquid. Excess hydrogel was then peeled/poured off the tissue. Following transfer into new 50 mL conical tubes, each brain section was submerged in an 8% SDS clearing buffer (20% SDS, 1xPBS, 0.01% sodium azide) and incubated in a 37°C shaking incubator for 48 h or until cleared. Brain tissue was divided into 1 mm thick coronal brain sections and optical clearance utilizing the CLARITY-PACT protocol[31].

### Immunofluorescent staining

Brain tissue was stained with human specific antibodies Stem121 (Y40410, 1:500, Takara Bio) and anti-nestin SP103 (ab105389, 1:100, Abcam) antibodies to locate and visualize IN administered LMNSC008 cells, as well as native tissue structure using NeuN (ab177487, 1:200, Abcam), GFAP (ab7260, 1:500, Abcam), and Tuj1 (ab18207, 1:750, Abcam) to mark mature neurons and glial cells. NeuN and Tuj1 were used as markers of mature and maturing neurons, and GFAP for reactive astrocytes. Tissue sections were placed in a 12-well plate and underwent three washes with TBST (1x TBS, 0.1% Triton X-100) for 10–15 min shaking at room temperature (RT). Sections were blocked (1xTBS, 5% BSA, 1% Triton X-100) for 1 h of shaking at RT, then incubated in primary antibody at RT, and covered from light for 24 h. After three 10-min washes in TBST, secondary antibody was added (ab150063, ab150062, ab150131, ab150106, 1:1000, Abcam), and the tissues were incubated for another 24 h at RT on a covered shaker. When applicable, a third set of washes was followed by 1 h of incubation in DAPI and a final fourth set of washes with TBS to prepare for mounting. Cleared, stained, and washed brain sections were mounted with RIMS solution (40g of Histodenz was diluted in 30 ml of 0.02M phosphate buffer, Sigma Aldrich), on glass slides with an attached 3-mm deep border. Slides were kept at RT and protected from light until ready for imaging, which was within one week of staining to prevent signal degradation.

### Imaging and quantification

Mounted slides were visualized and imaged using a ZEISS LSM900 confocal laser scanning microscope, and Zen (Blue) was used to set and capture these images. First, a large cross-sectional tile image of the brain section was created at 10X magnification to create a “map” of the subsequent z-stack images taken over the hippocampal and TBI regions of TBI and SHAM control brains. Boundaries were set, and 16 focus points were distributed, adjusted, and focused in a single light channel. Depending on the immunostaining used, the tile was set to capture each channel with appropriate exposure times in widefield by using the “set exposure” option. Signals for green fluorescence, AF647, AF555, and DAPI stains were captured using the 488nm, 640nm, 561nm, and 405nm lasers, respectively. Individual confocal Z-stacks at 10X magnification were taken along the areas of TBI and hippocampus when visible, as well as the uninjured contralateral hemisphere near the hippocampus (dentate gyrus) and cortical edges. The image size of a 2-dimensional section was 1024 pixels; scan speed was set to 8, bi-lateral direction, 2x averaging. All light channels were set to 1 Airy Unit and individually adjusted for master gain and diode laser intensity (%) as appropriate to maximize image output clarity Z-stack range was from 200–350 µm thick with a 7 µm interval depth. All other parameters were left at default settings.

Following imaging, Zen files were converted and uploaded to IMARIS, where the 3D sections were processed using the Spot tool following background subtraction in all color channels to correct for any autofluorescence in the tissue or background staining. The Spots tool, which uses an approximated average user-input average diameter to identify and count points of greatest light intensity in a single-color channel, was used to calculate the average stained cell count. A 2 µm range of cell sizes in 0.25 µm increments was used to get a distribution of the average count across a range of input diameters for GFAP-stained reactive astrocytes and NeuN-stained maturing neurons in coronal sections. Measurements were done in the two hippocampal dentate gyrus regions in both the right-TBI and left-normal hemispheres, as well as the TBI damage site in the right hemisphere and intact coronal areas of the respective contralateral hemisphere.

### IHC staining and cell quantification of macrophages

Brain tissue was processed for immunohistochemical (IHC) staining for rat myeloid M1 and M2 cells and for hematoxylin & eosin (H&E) staining to determine the fate of these cells in TBI and SHAM controls. Coronal sections (n = 200 per rat) from each treatment group (CCI + NSC, CCI + VEH, SHAM + NSC, and SHAM + VEH) were cut and prepared for paraffin slides. Every twentieth slide was H&E stained. Areas demonstrating TBI damage were stained with CD68 (ab283654 for M1, Abcam) and CD163 (ab182422 for M2, Abcam) antibodies. QuPath was used for IHC quantification of CD68 and CD163, with files uploaded in their native NDPI format from the Hamamatsu scanner. Slides were scanned at 20x and produced as brightfield images. The brain area was manually outlined using fill and draw tools. Slides were then analyzed using default positive cell detection parameters for optical density sum, though for CD163 the intensity threshold was modified to 0.85 while CD68 remained at default. Artifacts and areas of darkness due to tissue folding were manually removed by two independent technicians to limit false positive cell counting. For each rat, the hemisphere containing CCI-induced damaged tissue was compared to the contralateral, undamaged side. The main outputs of analysis were highlighted area, total cell count, and total positive cell count.

### Multiplex sandwich-based ELISA assays

Multiplexed ELISAs were performed on brains from each CCI group (CCI + VEH, CCI + NSC and SHAM + VEH). Quantibody^®^ Rat Cytokine Array 67 Kit consisted of a combination of 2 non-overlapping arrays (QAR-CYT-3 and QAR-CYT-4, RayBiotech) to quantitatively measure the concentration of 67 rat cytokines and it is suitable for tissue lysate samples. A subset of snap frozen brains from each treatment group were dissected (Fig. S7), tissue lysates were prepared using Lysis Buffer (EL-lysis, RayBiotech) and Protease Inhibitor (AA-PI, RayBiotech) and a hand-held tissue homogenizer (PRO-PK-01200PMGXL, Pro Scientific). Tissues were lysed in 1X solution at the highest setting (level 3) for 2 minutes, then centrifuged to aid in the separation of particulate matter left over from cell debris and portions of analysis of tissue from pure lysate. These lysates were packaged and shipped to RayBiotech Services, where the Quantibody ELISAs were performed. The impact of TBI on cytokine expression was evaluated by comparing tissue samples from the impacted TBI site to the non-impacted contralateral site (Table 1). The proteins that were most affected due to TBI and due to the treatment with NSCs were assessed by the analysis listed in Fig. S4 and S5. The average protein concentration at each site was calculated as the average of both rats per cohort. From these average protein concentrations, the ratio of TBI to contralateral site as well as treated to non-treated rat was calculated. The cytokine concentration ratio of CCI site to contralateral site of the untreated rats (CCI + VEH-R vs CCI + VEH-L) was evaluated to quantify the impact of CCI on cytokine expression. The same methods were used to analyze SHAM + VEH (L) vs SHAM + VEH (R) and compared as controls. The cytokine expression ratio of CCI site in treated rats was compared to the CCI site in untreated rats (CCI + NSC-R *vs* CCI + VEH-R) to quantify the impact of NSC treatment on the cytokine expression. Mathematically, it was done as below:

1 $$ChangeinCCIrelatedcytokines=\left[\frac{Averagecytokineconcentration_{CCI+VEH-R}}{Averagecytokineconcentration_{CCI+VEH-L}}-1\right]\times 100\%$$

2 $$Changeintreatmentrelatedcytokines=\left[\frac{Averagecytokineconcentration_{CCI+NSC-R}}{Averagecytokineconcentration_{CCI+VEH-R}}-1\right]\times 100\%$$

Where the average cytokine concentration at a site is calculated as an average over all the rats in that comparison group. For comparison with controls a similar change in cytokine expression of SHAM + VEH/NSC-R to SHAM + VEH/NSC-L was quantified.

### NanoString RNAseq sample preparation and data acquisition

Brain tissue was dissected, and RNA was isolated from snap frozen tissues according to a diagram in figure S7 and half was used for Nanostring analysis and half for protein ELISA assays. RNA expression in the ipsilateral and contralateral hemispheres of NSC and VEH-treated TBI brains was analyzed using the NanoString nCounter platform (NanoString Technologies) by digitally detecting and counting RNA expression in a single reaction without amplification. Each assay included six positive and eight negative RNA assay controls, plus ten mRNA housekeeping controls. RNA was hybridized with the Codeset from the gene panel at 65°C for 16 h. The post-hybridization probe-target mixture was quantified using the nCounter Digital Analyzer, and all data analyses were performed on nSolver (NanoString Technologies). All raw data were first normalized with internal positive and negative controls to eliminate variability unrelated to the samples, then normalized to the selected housekeeping genes using Geometric Means methods. Statistically significant (*p* < 0.05) and < 2-fold up- or down- regulated genes were analyzed and clustered as described below.

The datasets generated and analyzed during the current study are available in the [GSE242031] repository, https://www.ncbi.nlm.nih.gov/geo/query/acc.cgi?acc=GSE242031.

## Results

### IN administered NSCs migrate to hippocampal regions of the TBI brain

**Figure 1:**
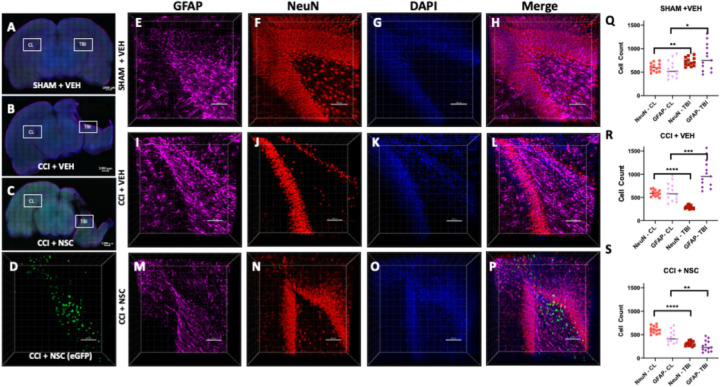
Visualization of intranasally administered NSCs in the injured neurogenic zone. **(A-C)**1 mm-thick coronal brain sections were stained for astrocytes (far-red), maturing neurons (red), and nuclei (blue). Location of z-stacks for contralateral (Fig. S1) hemispheres for each brain are labeled on 10X magnification cross-sectional tile images. (**D**) eGFP signal was only produced in TBI+LMNSC008 tissue. (**E-P**) 3D images of 250–350 µm-thick z-stacks taken at 10X magnification show individual and merged color channels as well as the localization of eGFP-labeled LMNSC008 cells at 39 days post-surgery from SHAM + VEH, -CCI + VEH, and CCI + LMNSC008 groups. (**Q-S**) Histograms of respective =IMARIS NeuN and GFAP quantifications in both the ipsilateral (impact) and contralateral (non-impact) hemispheres showing the average number of cells for various cell sizes. **(Q) SHAM +VEH:** NeuN expression in TBI vs CL hemisphere using unpaired t test p<0.002; GFAP p<0.01. **(R) CCI + VEH**: NeuN expression in TBI vs CL hemisphere p <0.0001; GFAP p<0.0005. **(S) CCI + NSC:** NeuN in TBI vs CL p<0.0001; GFAP p<0.001. Data was analyzed using unpaired t test and two-tailed p values have shown.

To examine whether migration and distribution of NSCs is specific to TBI sites, we performed CLARITY-PACT tissue clearing in combination with 3-dimensional (3D) confocal microscopy using 1mm think coronal brain slices overlapping the TBI site. Our extensive data analysis of multiple brain slices and confocal 3D imaging revealed the presence of fluorescent LMNSC008.eGFP cells surrounding damaged tissue and in the dentate gyrus, which indicates that NSCs were delivered to the brain and were present in the neurogenic areas at the TBI site, specifically in CCI + NSC treatment group (Fig. 1D). The contralateral hemisphere hippocampus of all brains showed no green eGFP expressing cells (Fig. S1), suggesting that NSCs travel in a brain injury site-specific manner and migrate to neuroregenerative areas of in the injured hemisphere (Fig. 1). Additionally, distinct staining of mouse NeuN and GFAP and dense areas of green fluorescence suggest the presence of eGFP-expressing NSCs in specimens of the CCI + NSC group, but not detected in CCI + VEH or SHAM + VEH groups (Fig. 1H, L, P). Figure 1 shows the localization of several fluorescently labeled LMNSC008.eGFP cells to neurogenic zones in the dentate gyrus of CCI + NSC rats (Fig. 1P). There is a lack of such a presence of NSCs in sham-surgery brains as well as in the contralateral hemisphere of TBI-model rat brains. Z-stack images were taken in both hippocampal zones of the ipsilateral (injured) and contralateral (non-injured) hemispheres of the female rat brain in SHAM + VEH, CCI + VEH, and CCI + NSCs groups. The migration of LMNSC008.eGFP cells was only detected in ipsilateral hippocampal regions. NSCs were absent from both contralateral (CL) hemisphere hippocampal zones and in other treatment groups (green channel not shown, CL hemisphere images in Fig. S1). Immunofluorescent staining of the cleared brain tissue sections and subsequent quantification of NeuN (neural – red stain) and GFAP (glial – far red stain) using the IMARIS Spot tool at various cell size estimates per stain shows a decrease in abundance of NeuN stain in the TBI hemispheres of CCI brains compared to the CL hemisphere (Fig. 1Q,R,S). While the sham surgery brains show little difference between VEH and NSC-treated groups, the TBI site of CCI brains demonstrated a greater GFAP staining in the TBI hemisphere compared to the CL hemisphere. Since GFAP is a by-product of scar tissue formation by reactive astrocytes, an increase in its expression is usually correlated with increased immune and inflammatory response [32]. CCI + NSC brains were the only group displaying a decrease in GFAP expression in the TBI hemisphere as compared to the CL hemisphere. This strongly suggests that the inflammatory response responsible for harmful long-term effects after CCI is reduced by administration of LMNSC008 cells sometime before or during the 39th day following TBI in the female brain. These results aligned with a decrease of inflammatory cytokines in damaged (TBI) to undamaged (CL) hemispheres of the brain in our data to further support our hypothesis for the improved recovery from the TBI following treatment with LMNSC008 cells (Fig. 3B).

### Intranasally Administered LMNSC008 Cells Migrate Specifically towards TBI sites

**Figure 2:**
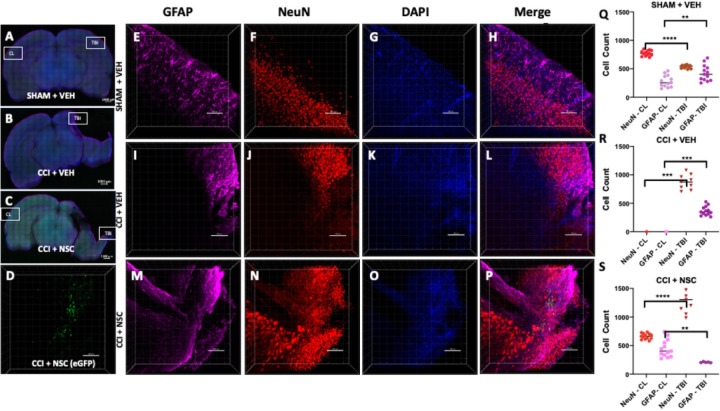
Visualization of NSCs in the damaged areas of the female rat TBI after intranasal administration. **(A-C)** Staining of astrocytes (far red), maturing neurons (red), and nuclei (blue) on 1 mm-thick coronal rat brain sections in TBI and Cortical regions of the brain. **(E-P)** 3D images of Z-stacks taken at 10X magnification through 250–350 µm show individual and merged color channels as well as the localization of eGFP-labeled LMNSC008 NSCs 39 days post-surgery of three female rat brains from groups: SHAM + VEH, CCI + VEH, and CCI + NSC. **(Q, R, S)** Quantifications of NeuN and GFAP staining in both the TBI and CL hemispheres. **(D)** Green eGFP signal was only seen in the CCI + NSC brain section. **(Q) SHAM +VEH:** NeuN expression in TBI vs CL hemisphere using unpaired t test p<0.0001; GFAP p<0.0065. **(R) CCI + VEH**: NeuN expression in TBI vs CL hemisphere p <0.0002; GFAP p<0.001. **(S) CCI + NSC:** NeuN in TBI vs CL p<0.0001; GFAP p<0.001. Data was analyzed using unpaired t test and two-tailed p values have shown.

Furthermore, we investigated the TBI site-specific distribution of LMNSC008 cells stably expressing eGFP as visualized by confocal microscopy (Fig. 2D). We performed CLARITY-PACT tissue clearing and confocal imaging using 1 mm coronal brain sections prepared across the CCI injury and CL sites to concurrently show images of both the CCI injury site in the right hemisphere and cortical areas of the undamaged CL hemisphere. Immunofluorescent staining of mouse NeuN and GFAP was performed, and the tissue was additionally contra-stained with DAPI. Z-stack images were obtained similarly at multiple TBI and CL sites (Fig. 2A, B, C and Fig. S2). Single channel staining of GFAP, NeuN, and DAPI was performed on multiple coronal sections (1 mm brain sections S1 to S7) and 3D images of a 1 mm section for SHAM + VEH (Fig. 2E-H), CCI + VEH (Fig. 2I-L), and CCI + NSC (Fig. 2M-P) groups. Quantification was done where possible, which indicated a decrease of GFAP in the cortical regions in the CCI + NSC group as compared with CCI + VEH or SHAM + VEH groups (Fig. 2Q, R, S). TBI-site LMNSC008.eGFP cells were only visualized in TBI + NSC treated rats (Fig. 2D).

### Quantitative Assessments of Inflammatory Cytokines in NSC-treated vs Non-treated CCI Rats

**Figure 3:**
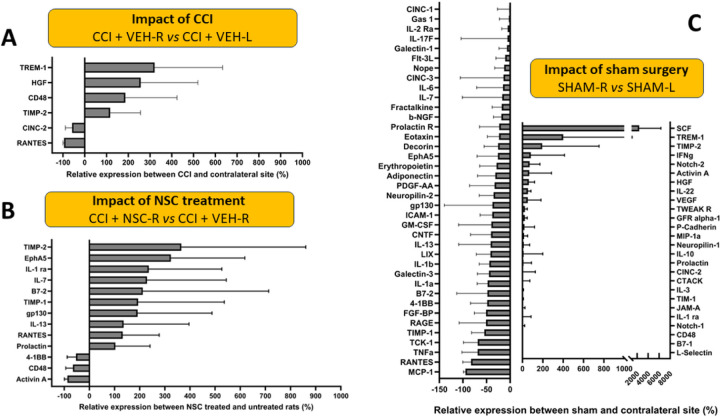
Cytokine ELISA results from female rat brain lysates at contralateral [CL-left] and CCI [TBI-right] hemispheres. **(A)** Cytokine expression comparing TBI sample (right hemisphere) to CL sample (left hemisphere) showing the impact of TBI on cytokine expression levels. **(B)** Cytokine expression levels when comparing the TBI hemisphere samples of treated rats to the untreated rats showing the impact of NSCs on cytokine expression levels. Only cytokines for which the change is either greater than 100% or less than −50% are shown. Figures S4 and S5 show all cytokine data analyzed. **(C)** Cytokine expression comparing the right and left hemispheres of sham surgery rats (both VEH and NSC groups were included in this analysis).

A subset of snap frozen brain from each treatment group was used for tissue ELISA analysis. Tissue dissection is schematically described in Fig S7. Two lysates were prepared from each rat brain (left and right), where one manually dissected section contained the area around the left hippocampus, and the other the area of the right hippocampus, which was or was not impacted by a CCI. Both protein analysis using ELISA and gene expression using Nanostring assay were performed on the same tissues, split in half and snap frozen in liquid nitrogen (Fig S7). ELISA experiments were performed in quadruplicates and standard curves were generated using a standard cytokine mix. From that quantification data, graphs showing the impact of TBI (Fig. 3A) and impact of NSC treatment (Fig. 3B) were generated to understand 1) the differences in protein expression between the left and right hemisphere when a TBI is present, and 2) the differences in protein expression between a TBI brain that has received or not received an NSC treatment. We demonstrate overall increase in cytokines at the TBI sites when compared with contralateral (Fig. 3A). Furthermore, we compared the impact of NSC treatment and demonstrated a ratio of CCI + NSC-Right vs CCI + VEH-Right (Fig. 3B). The reliability of a cytokine to reflect the biology is high only if all rats [from the same treatment group] show an increase or both show a decrease in the cytokine expression when compared. Thus, only the cytokines for which all rats showed similar trends (i.e., both increased or both decreased) in expression when comparing the two sites were considered for comparison. The lowest reliable estimate of the protein concentration by the instrument is the limit of detection (LOD) for that protein. Proteins whose concentrations at both the TBI site and the contralateral side for all rats (CCI + VEH and CCI + NSC) were less than the LOD were not considered for analysis. Thus, to calculate the ratio of protein expression, the protein concentrations that were less than the LOD were replaced by the LOD (so as to not divide by cytokine concentrations that are numerically zero and to deal consistently with proteins whose expression was lower than instrument LOD).

Quantification of infiltrating CD68 and CD163 macrophages was performed after IHC staining of coronal brain sections in the TBI and CL hemispheres. We demonstrated significantly higher staining of both macrophage markers CD68 and CD163 in the TBI groups, regardless of whether treated with NSC or VEH, relative to the SHAM controls. SHAM controls showed no difference in CD68 or CD163 staining in either hemisphere (Fig. S3I). QuPath software was used to quantify DAB staining for CD68 and CD163 in paraffin-embedded coronal rat brain sections derived from the four treatment groups (CCI + NSC; CCI + VEH; SHAM + NSC; SHAM + VEH) (Fig. S3A-H). Staining of the TBI hemisphere and CL hemisphere were quantified separately for each rat after scanning and identifying the tissue area in QuPath (right and left hemispheres). Results from each group were averaged by mean and graphed for female rats (Fig. S3I). There was a greater increase of CD68 staining in the TBI hemisphere when the NSC treatment was given to CCI rats than when the VEH treatment was administered. For CD163 staining, TBI and CL hemispheres responded more similarly to another within the TBI + NSC group, with a slight increase in cell count in that group than in other groups. The data indicate greater M1 and M2 macrophage activity in the damaged vs. uninjured region.

**The angiogenesis ELISA** has the capacity to show whether NSC treatment might influence the production of proteins involved in the production of new blood vessels in the rat brain, and by proxy the development of new neural networks in recovering tissue (Fig.S6). **The cytokine ELISA** was chosen to demonstrate how NSC treatment to the CCI brain alters the immune response, to draw parallels between these results and those of the M1/M2 profiling experiments prior (Fig. S4 and S5).

#### Differential gene expression between NSC treated and untreated groups

**Figure 4:**
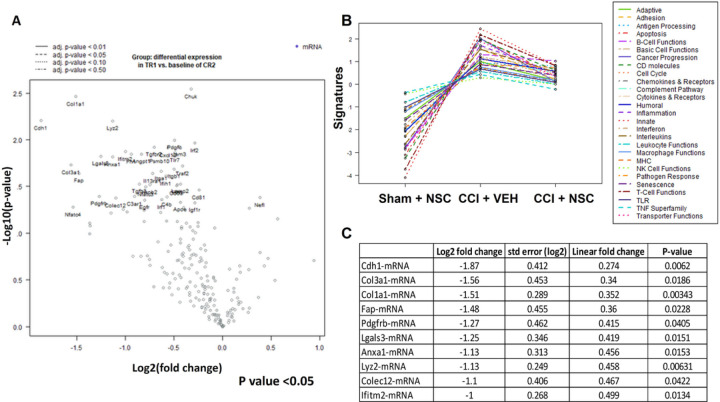
Differential gene expression data for CCI + VEH vs. CCI + NSC from TBI and contralateral hemispheres. (**A**) Volcano plot showing comparing differences in gene expression between CR2 (CCI + VEH) and TR1 (CCI + NSC). Each dot represents a gene within the comparison performed. (**B**) Pathway analysis of CR1 (SHAM + VEH), CR2 (CCI + VEH) and TR1 (CCI + NSC). **(C)**Table of top 10 upregulated genes in CR2 (CCI + VEH) versus TR1 (CCI + NSC).

Snap frozen brain tissues dissected from left (CL) and right (TBI) hemispheres were also used for NanoString analysis (Fig. S7) in order to detect differentially expressed genes in CCI + VEH and CCI + NSC groups in the TBI hemisphere (Fig. 4). The brain tissue collection schema is shown in Fig S7 and the sample attribute file is shown in Table S1.

NanoString pathway analysis revealed the top genes that were up- or down- regulated in CCI + VEH and CCI + NSC groups (Fig. 4A). The quantification of expression for 700 genes in female rats which either had (TR1 [CCI + NSC]) or had not (CR2 [CCI + VEH]) received NSC treatments after CCI is quantified at the right hemispheres (TBI site) in a volcano plot (Fig. 4A). Nanostring nSolver analysis revealed that gene expression of CR2 [CCI + VEH], TR1 [CCI + NSC] tissues demonstrated different gene expression profiles, demonstrating downregulation of multiple genes and pathways in the TR1 group when compared with CR2 (Fig. 4B, right, Table S1). Among pathways that were differentially expressed at CCI + VEH, CCI + NSC treatment altered genes involved in leukocyte functions (score 2.6), genes involved in extracellular matrix remodeling and CD molecules (1.9), interferon signaling (1.9). NSC treatment reduced genes and pathways involved in inflammatory response (1.8), such as microglial function, cytokines, and receptors, which were downregulated in CCI + NSC treatment group as compared with CCI + VEH. Furthermore, cell type analysis revealed downregulation of DC, macrophages, and CD45 cells at CCI + NSC vs CCI + VEH groups (data not shown). A list of the 10 top upregulated genes in CR2 (CCI + VEH-right) versus TR1 (CCI + NSC-right) groups (n = 4) with cut off 2 and − 2 fold change and p < 0.05 are shown in Fig. 4C. Among the genes activated in CCI + VEH group are Cdh1 (contributes to TBI-induced inflammation, and inhibition of Cdh1 contributes to anti-inflammatory treatment for TBI)[33], collagen and fibroblast activation genes (Col3a1, Col1a1, Fap)[34, 35], and Lyz2, which is a potential target for novel strategies for treatment of TBI that involve innate immune response. PDGFRb is another target for the treatment of acute TBI in rats, and activated PDGFRb participates in secondary brain injury after TBI. Interventions to target PDGFRb have been applied to improve outcomes after TBI, and PDGFRb was downregulated by NSC treatment[36]. These data suggest a beneficial multifactorial effect of NSCs on genes and pathways involved in inflammatory response, such as microglial function, cytokines, and receptors, which are downregulated after NSC treatment. The data demonstrate a robust proof-of-concept for IN administered LMNSC008-mediated therapy for TBI and provides a strong rationale for IN delivery for translation in TBI patients.

## Discussion

Human neural stem cells (NSCs) are attractive candidates to help restore brain function through repair (neuroprotection) or reconstruction (cell replacement) after brain trauma. Our working idea is that NSCs, through their intrinsic migration to sites of brain inflammation, promote an environment that fosters cellular regeneration by mechanisms such as delivering growth factors, suppressing inflammation, reducing axonal injury, and/or differentiating into mature neural-lineage brain cells. Supporting the feasibility of NSCs as a potential treatment option for TBI, NSCs are currently being evaluated in clinical trials for repair of damage associated with stroke and multiple sclerosis[33–42]. Human NSC grafts have also been assessed as a cellular therapy for cell replacement in Pelizaeus-Merzbacher disease, a rare CNS leukodystrophy, and other neurodegenerative diseases[43–53]. Allogeneic NSCs derived from human fetal brain tissue and immortalized using the C-MYC gene were also successfully tested in a clinical trial for use after stroke, demonstrating initial safety in humans and a potential therapeutic effect (Reneuron Limited, UK; NCT02117635)[33, 54–57]. However, a major obstacle to the clinical translation of NSC-based therapy is optimizing cell delivery to areas of injury in the CNS to achieve a therapeutic effect[14, 58–60]. Although intravenously injected NSCs can cross the BBB and localize to damaged tissue, they show limited accumulation in the brain (less than 1% of injected NSCs)[61]. Furthermore, intravenous administration of NSCs can lead to immune responses and other systemic complications[61]. Intracranial administration of NSCs avoids potential systemic reactions and results in engraftment of 5–15% of injected cells at the intended target site[61]. Unfortunately, intracranial administration is invasive, costly, and potentially damaging to normal tissue. In the proposed studies, we build on our previous work using a well characterized and immortalized human NSC line that stably expresses the L-MYC gene (LMNSC008)[12, 28]. Following intranasal administration in rats, these LMNSC008 cells demonstrated migration from the intranasal cavity towards sites of pathology resulting from CCI brain injury, as a model of TBI[20]. LMNSC008 cells administered into rats (immunocompetent) models (with defined dose and schedule of administration) demonstrated migration, engraftment, neuroprotective effect, and lack of tumorigenicity in TBI models. Furthermore, the results of this study demonstrate migration of LMNSC008 cells to the injury sites in the hippocampus after repeated IN administrations. Additionally, we demonstrated that presence of NSCs in the TBI promotes tissue regeneration, as measured by ELISA and Nanostring analysis, and can serve as a therapeutic candidate for NSC-based therapy after TBI.

Successful translation of IN administration of therapeutic NSCs will be more cost-effective, non-invasive, and safer than current approaches of cellular therapies because it can be done in an outpatient setting and can overcome the risks and bottlenecks associated with intravenous and intracranial administration. Thus, development of cellular therapy of therapeutic NSCs delivered IN represents a promising method for treating patients with a wide range of TBI brain injury and other pathologies of CNS.

## Supplementary Material

Supplement 1
