# Supplementary material for: Neuroprotective potential of intranasally delivered L-myc immortalized human neural stem cells in female rats after a controlled cortical impact injury
Source: Res Sq. 2023 Sep 5:rs.3.rs-3242570. Preprint. [Version 1] doi: 10.21203/rs.3.rs-3242570/v1 (PMC10503851; doi:10.21203/rs.3.rs-3242570/v1)
Supplement: Supplement 1 [file NIHPPRS3242570V1-supplement-1.pdf]

Supplementary Files

This is a list of supplementary files associated with this preprint. Click to download.

- AmirbekyanSupplementaldataFinal.docx
- AmirbekyanTable1SR.png.jpg
